# A Subpopulation of the K562 Cells Are Killed by Curcumin Treatment after G2/M Arrest and Mitotic Catastrophe

**DOI:** 10.1371/journal.pone.0165971

**Published:** 2016-11-10

**Authors:** Macario Martinez-Castillo, Raul Bonilla-Moreno, Leticia Aleman-Lazarini, Marco Antonio Meraz-Rios, Lorena Orozco, Leticia Cedillo-Barron, Emilio J. Cordova, Nicolas Villegas-Sepulveda

**Affiliations:** 1 Departamento de Biomedicina Molecular, Centro de Investigación y de Estudios Avanzados, Mexico City, Mexico; 2 Laboratorio de Inmunogenómica y Enfermedades Metabólicas, Instituto Nacional de Medicina Genómica, Secretaria de Salud, Mexico City, México; Universita degli Studi di Palermo, ITALY

## Abstract

Curcumin is extensively investigated as a good chemo-preventive agent in the development of many cancers and particularly in leukemia, including treatment of chronic myelogenous leukemia and it has been proposed as an adjuvant for leukemia therapies. Human chronic myeloid leukemia cells (K562), were treated with 20 μM of curcumin, and we found that a subpopulation of these cells were arrested and accumulate in the G2/M phase of the cell cycle. Characterization of this cell subpopulation showed that the arrested cells presented nuclear morphology changes resembling those described for mitotic catastrophe. Mitotic cells displayed abnormal chromatin organization, collapse of the mitotic spindle and abnormal chromosome segregation. Then, these cells died in an apoptosis dependent manner and showed diminution in the protein levels of BCL-2 and XIAP. Moreover, our results shown that a transient activation of the nuclear factor κB (NFκB) occurred early in these cells, but decreased after 6 h of the treatment, explaining in part the diminution of the anti-apoptotic proteins. Additionally, P73 was translocated to the cell nuclei, because the expression of the C/EBPα, a cognate repressor of the *P73* gene, was decreased, suggesting that apoptosis is trigger by elevation of P73 protein levels acting in concert with the diminution of the two anti-apoptotic molecules. In summary, curcumin treatment might produce a P73-dependent apoptotic cell death in chronic myelogenous leukemia cells (K562), which was triggered by mitotic catastrophe, due to sustained BAX and survivin expression and impairment of the anti-apoptotic proteins BCL-2 and XIAP.

## Introduction

Chronic myeloid leukemia is characterized by the increased growth of myeloid lineage cells and their accumulation in blood and bone marrow. Approximately 95% of the cases are characterized by clonal expansion of myeloid cells containing the Philadelphia chromosome [1, 2] which has a translocation of chromosomes 9 and 22 t(9;22), producing a fusion between the *BCR* and *ABL* genes [3]. The resultant BCR-ABL hybrid protein is a constitutively active tyrosine kinase that functions as an oncoprotein; consequently, it activates several important signal transduction pathways involved in cell growth inhibition of cellular differentiation and programmed cell death [4]. Although, several tyrosine kinase inhibitors targeting the BCR-ABL hybrid have been developed and shown to be successful for chronic myeloid leukemia treatment, leukemia cells can become resistant to treatment [5]. This likely due to a small population of highly quiescent chronic myelogenous leukemia cells, which are insensitive to the tyrosine kinase inhibitors and they are believed to be early leukemia progenitor cells [6–8]. However, the precise molecular events resulting in cell resistance to therapeutic drugs have not been completely elucidated [8, 9].

Although new tyrosine inhibitor derivatives have been reported to have higher efficiencies in the treatment of chronic myelogenous leukemia; a reduced number of the patients will progress to the accelerated phase of the disease, the most aggressive illness form, namely blast crisis [9, 10] and eventually these patients may die. Therefore, the use of some polyphenolic compounds as supplements or adjuvants for chemotherapy in chronic myelogenous leukemia and other types of leukemia has been extensively researched, for example, curcumin and its chemical derivatives.

Curcumin (diferuloylmethane) is a biphenolic compound extracted from rhizomes of *Curcuma longa* plants; it is the major component of the tumeric spice. Multiple studies have shown that it has several biological functions, including anti-oxidant, anti-inflammatory and anti-tumor properties. In the last decade, curcumin has been proposed as adjuvant for leukemia therapies, including treatment of chronic myelogenous leukemia. In fact, curcumin has shown to inhibit the growth, as well as metastases in several tumor derived cell lines [11]. In addition, a curcumin derivative induces apoptosis in treatment resistant chronic myelogenous leukemia [12]. In a previous study, a mouse model of myelogenous progenitor cells with exogenous expression of the *BCR-ABL* chimeric gene in the 32D cells was assessed, and 20 μM of curcumin produced cell cycle arrest in the G2/M phase, as well as changes in nuclear morphology typical of mitotic catastrophe. Cell death occurs in a caspase-dependent manner after multiple changes, including abnormal chromatin organization, multipolar chromatin segregation and cytokinesis defects [13]. Mitotic cell death is produced by an apoptosis-dependent or independent mechanism [14]. Apoptosis is a type of programmed cell death that plays an important role in maintaining cellular homeostasis; it is activated in response to severe DNA damage, bacterial or viral infections and eventually results in elimination of the damaged cells. During apoptosis, several cysteine-aspartate proteases, known as pro-caspases, are proteolitically processed and activated; the activated proteases are named caspases; they target many proteins involved in maintaining the cell function and integrity [15]. However curcumin has different effects depending on the cell types and research models [16]. Therefore, we aimed to investigate whether a human leukemia derived cell line containing the Philadelphia chromosome, is affected by curcumin in a similar manner, as the 32D progenitor mouse model.

A human chronic myelogenous leukemia cell line (K562) was treated with 20 μM of curcumin, and we found that a subpopulation of these cells were arrested and accumulate in the G2/M phase of the cell cycle. Further characterization of this cell subpopulation showed that after 18h of the treatment, the arrested cells displayed nuclear morphology changes resembling those described for mitotic catastrophe. Mitotic cells presented abnormal chromatin organization, collapse of the mitotic spindle and abnormal chromosome segregation. Then, cell death occurred in an apoptosis dependent manner along with a decrease in the levels of B-cell lymphoma 2 protein (BCL-2) and X-linked inhibitor of apoptosis protein (XIAP). Notably, expression levels of the two proteins decreased in a caspase-independent manner. Moreover, our results also showed that nuclear factor κB (NFκB) classical activation occurs early in the treated cells and decreased after 6 h of the treatment, explaining in part the decrease in the anti-apoptotic proteins. Additionally, P73 was translocated to the cell nuclei, and the expression of the C/EBPα, a cognate repressor of the *P73* activation, was reduced in the curcumin-treated cells, suggesting that apoptosis is triggered by elevation of the P73 protein acting in concert with the diminution of the two anti-apoptotic molecules.

## Materials and Methods

### Cell culture

The chronic myelogenous leukemia K562 cell line was obtained from the American Type Culture Collection (ATCC^®^ CCL-243^™^). Cells were grown at 37°C and 5% CO_2_ in RPMI supplemented with 10% fetal bovine serum, 50 μg/ml streptomycin, 50 U/ml penicillin and 1% (v/v) non-essential aminoacids, using a humidified incubator. For the experiments, all cells were grown to a density of 250,000 cells/ml in six-well or twelve-well culture plates and treated with 20 μM curcumin (Sigma Chemical Co, USA) for 12, 18 or 24 h. Control cells were treated with 0.1% Dimethyl sulfoxide (DMSO) from Sigma Chemical Co. The curcumin dosage was chosen on the basis of dose-response curves. Cell culture media, fetal bovine serum, antibiotics and molecular biology reagents were purchased from Gibco BRL (Invitrogen, USA). Z-VAD-FMK was purchased from R&D Systems (Minneapolis, MN, USA).

### Western blot and immunofluorescence assays

Protein extracts for the western blot analysis were prepared using 2–4 x 10 ^6^ cells. Cells were washed with phosphate-buffered saline (PBS) and lysed in 80–100 μl of lysis buffer (ProteoJet TM Mammalian Cell Lysis Reagent, K0301; Fermentas) that was supplemented with protease (Complete, Roche, DE) and phosphatase cocktail inhibitors (Phosphostop, Roche). Extracts were clarified by centrifugation for 20 min at 16,000 g at 4°C. The protein concentrations were determined using Bradford protein assay kit (Bio-Rad, USA). Soluble protein extracts (30 μg) were mixed with an appropriate amount of 2X or 4X Laemmli sample buffer, boiled, separated by SDS-PAGE and subsequently transferred to polyvinylidene difluoride (PVDF) membranes (PerkinElmer, USA). Membranes were blocked with 5% nonfat dry milk in Tris-buffered saline 1X supplemented with 0.1% Tween-20 and probed overnight using an appropriate primary antibody; 1:1000 dilutions were used for most primary antibodies, except for anti-actin (1:500) and anti-GAPDH (1:5000). For detection, 1:5000 dilutions of anti-rabbit (Invitrogen, Thermo Scientific, MA, USA), anti-mouse (Invitrogen) antibodies or anti-goat (Thermo Scientific, MA, USA) conjugated to horseradish peroxidase were used. Finally, the immunocomplexes were developed using SuperSignal West Pico or West femto Maximum sensitivity substrate (Thermo Fisher Scientific, MA USA) with a ChemiDoc (BioRad), imaging capture system. Most PVDF membranes were stripped and re-probed for actin. Nuclear fractions were prepared using 4 x 10^6^ cells and nuclear and cytoplasmic proteins extraction kit (NE-PER from Thermo Fisher Scientific, MA, USA) according to the manufacturer instruction. For immunofluorescence assays, 2 × 10^5^ cells were grown on cover slips treated with poli-L-lysine in twelve-well plates with 2.0 ml of RPMI-1640 supplemented with 10% FBS. Cells were harvested, washed using 1X PBS then fixed with 4% paraformaldehyde. Cells were stained or immunodecorated using the following conditions: For mitotic spindle staining, fixed cells were permeabilized with 1X PBS plus 0.1% Triton X-100 and blocked for 15 min in 1X PBS supplemented with 0.5% gelatin and 1.5% Fetal bovine serum, followed by incubation for 1h with anti-α-tubulin (1:500) and 45 min with an anti-mouse antibody coupled to FITC (1:25) (Jackson Immunoresearch). For caspase-3/p-H3S10 staining, cells were permeabilized with 0.25% Triton X-100 in 1X PBS and blocked 30 min in 1X PBS plus 2% BSA. Slides were incubated for 1 h with anti-H3S10p (1:50) and subsequently incubated with anti-rabbit coupled FITC (1:100) (Jackson Immunoresearch, PA, USA). For staining of active caspase-3, cells were incubated with sulfo-rhodamine coupled caspase-3 inhibitor (Red-DEVD-FMK, Calbiochem), in 1:100 dilution in PBS. For actin staining, cells were incubated with 0.06 U of Rodamine-Phalloidin (Invitrogen, Carlsbad CA, USA) in PBS. For nuclear staining cells were incubated with 1 μg/ml 4', 6-diamino-2-fenilindol (DAPI stain, Invitrogen). Slides were mounted using Vecta Shield (Roche) and visualized in a confocal microscopy, Olympus FV-300.

### Antibodies

Antibodies for survivin (# 2808), BCL-2 (# 2872), XIAP (# 2042), caspase-9 (# 9502), caspase-8 (# 9746), caspase-3 (# 9662), C/EBPα (# 2295), C/EBPβ (# 3087) and PARP (# 9542) were purchased from Cell Signaling; anti-BAX (336400) was supplied by Invitrogen, and anti-GAPDH was provided by Genetex (GTX627408); antibodies for P73 (sc-7957), hnRNPA1 (sc-10029), p-H3S10 (sc-8656-R) and α-tubulin (sc-32293) were obtained from Santa Cruz Biotechnology. The antibody against actin was a generous gift from Dr. M. Hernandez, Cinvestav-IPN, Mexico. Commercial secondary anti-mouse (115-095-008) and anti-rabbit IgG (111-095-003) conjugated with Fluorescein (FITC) were purchased from Jackson Immunoresearch.

### Cell cycle, viability and apoptosis assays

For cell cycle assays, 1 × 10^6^ treated K562 and control cells were fixed overnight at -20°C, using 70% ethanol solution, then they were recovered and washed with 1X PBS, and incubated with a 0.2–0.5 mg/mL RNase A for 1 h at 37°C. The cell suspension was supplemented with 10 μg/mL of propidium iodide, incubated on ice for 30 min and the samples were protected for light. Samples were analyzed using flow cytometry analysis (FACS BD LSR Fortessa, Becton Dickinson). The relative fluorescence value for 2N DNA indicated G1 cell population and 4N DNA indicated the G2/M cell population. For the viability assays, K562 cells were stained with 1 μl of the Texas Red fluorescent dye (λ_ex_ = 595 nm and λ_em_ = 615nm) from the LIVE/DEAD^®^ Fixable Dead Cell Stain (L23102; Invitrogen), as recommended by the manufacturer. Red-stained cells (2 × 10^4^ cells) were counted using the FL-2 channel, of the FACS Calibur flow cytometer (Becton Dickinson).

For analysis of apoptosis, DNA ladder fragmentation or TUNEL protocol were used. Briefly, 5–7 × 10^6^ cells were washed with 1X PBS and pelleted by centrifugation to 1000 g; subsequently, the cells were suspended in lysis buffer (0.2% Triton X-100; 10 mM Tris-HCl, pH 7.4, 10 mM EDTA) and incubated for 15min on ice; then the cells were centrifuged at 12000 g for 20 min and the supernatant was recovered and incubated for 1 h at 37°C with 100 μg/ml of DNase free RNase A. DNA extraction was performed by adding one equal volume of phenol: chloroform: isoamyl alcohol (25:24:1), and one step of chloroform:isoamyl alcohol (24:1) extraction. DNA fragments were pelleted, by adding 300 mM NaCl and 2.5 volumes of 100% ice-cold ethanol followed by incubation overnight at –20°C and subsequent centrifugation. Then, the samples were washed with 1 ml of 70% ethanol. DNA was suspended in 1X TE buffer [17]. Samples were mixed with 6X DNA loading buffer and visualized by 1.5% agarose gel electrophoresis.

For TUNEL assays, K562 cells were stained using an APO-BrdU™ TUNEL Assay Kit (Molecular Probes, Invitrogen), according to manufacturer recommendations. Briefly, 1 × 10^6^ cells were fixed with 1% paraformaldehyde for 15 min on ice, centrifuged and incubated overnight in 70% ethanol at -20°C. Later, the cell pellet was recovered by centrifugation and washed and the samples were incubated with 50 μl of DNA-labeling solution according to the manufacturer's protocol. Finally, to determinate the DNA content, each sample was suspended in propidium iodide/RNase A staining buffer and incubated for 30 min at room temperature. Samples were analyzed with FACS Calibur flow cytometer and at least 10000 events were captured and analyzed with Summit software (Beckman Coulter, Brea, CA USA).

For Detection of p-H3S10 levels, curcumin treated K562 cells and controls were recovered and fixed with 70% ethanol at –20°C overnight. Then cells centrifuged at 1400 g centrifugation, permeabilized with PBS plus 0.25% Triton X-100 on ice for 15 min and incubated with 0.5 μg of p-H3S10 antibody diluted in 1X PBS supplemented 1% BSA for 2 h at room temperature with agitation. Subsequently, the cells were washed with PBS plus 1% bovine serum albumin, and incubated with anti-rabbit- coupled to FITC (1:200) for 30 min at room temperature. To determinate the DNA content, cells were incubated with 0.5 mg/ml of DNase free RNase A for 30 min at 37°C, and 10 μg/ml of propidium iodide for 15 min at 4°C [18]. For caspase-3 staining, cells were incubated with antibody against p-H3S10 for 1 h, followed by incubation with anti-rabbit-coupled Alexa 594 (Invitrogen) diluted 1:8000 in 1X PBS, 1% BSA. Finally the cells were incubated with a sulpho-rhodamine-coupled caspase-3 inhibitor (Red-DEVD-FMK, Calbiochem) diluted 1:200 in 1X PBS plus 1% bovine serum albumin, for 30 min at 37°C. Samples were evaluated using BD LSR Fortessa flow cytometer (Becton Dickinson, Franklin Lakes, NJ, USA), at least 20000 events were captured and analyzed with Summit software (Beckman Coulter).

### Statistical analysis

Results are expressed as means ± s.d. of at least three independent experiments. Differences between control and curcumin treatments were analyzed by one-way analysis of variance followed by Tukey’s multiple comparison tests. Differences were considered significant at P < 0.05. Statistical analysis was done with the GraphPad Prism 5.0 Software (Hearne Scientific, Pty Ltd, Melbourne, Australia).

## Results

**Fig 1:**
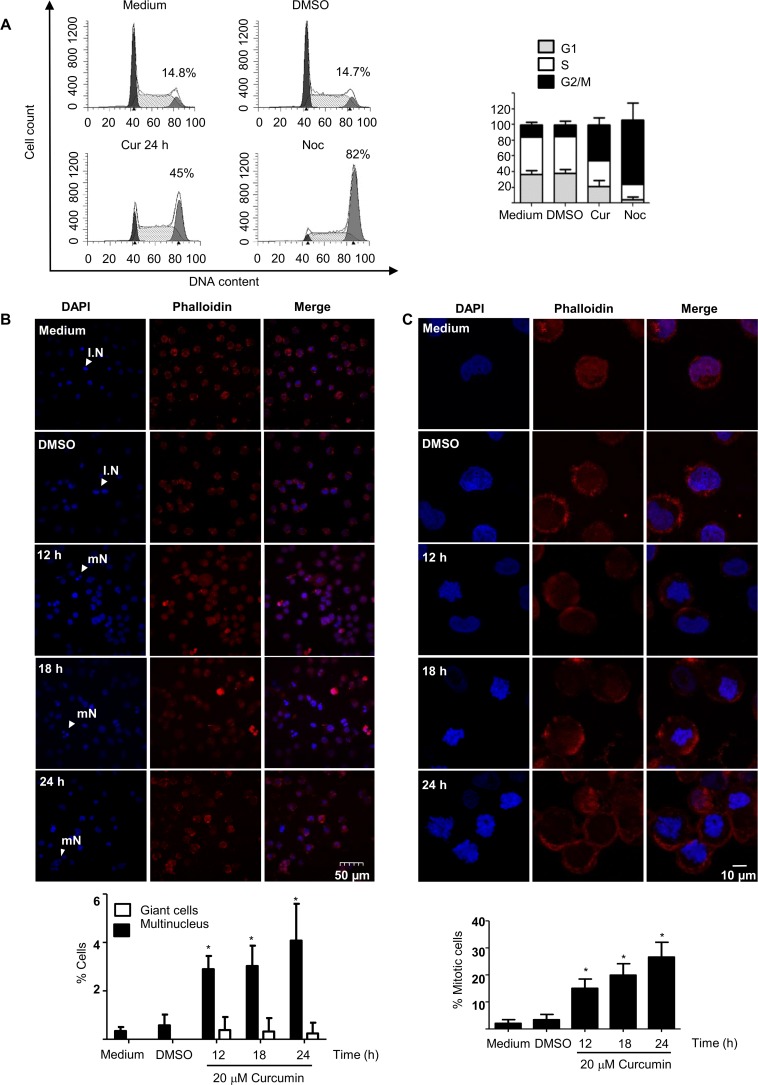
K562 cells treated with curcumin were arrested in G2/M2 phase. **A)** A subpopulation of the curcumin-treated K562 cells was arrested in G2/M phase, as determined by FACS analysis. Cells were stained with DAPI or immunostained with rhodamine-phalloidin (red fluorescence) to visualize the nuclei and actin fibers, respectively. **B**) Subsequently, they were analyzed by immunofluorescence microscopy, and the percentage of giant or multinucleated cells was determined. **C**) The amplification of these cells clearly showed the condensed chromatin and the actin cytoskeleton changes due to early mitotic events. Condensed nuclei were considered an indirect measure of mitotic cells. As a positive control for the enriched G2/M population, K562 cells were treated for 24 h with 100 nM nocodazole (Noc). IN = interphase nucleus; mN = multinucleated cell.

Previous results in our group indicated that concentrations higher than 15 μM curcumin induce G2/M in a fraction of the K562 cells. These results were consistent with data indicating that mouse 32D cells, which expressed exogenous *BCR-ABL* chimeric gene were arrested during the G2/M phase after incubation with 20 μM of curcumin [13]. The treatment of the K562 cells with concentrations of 15–20 μM of curcumin resulted in enrichment of a fraction of cells arrested in G2/M, and at 20 μM of curcumin, higher accumulation of the arrested cells was observed (Fig 1A). Therefore, to characterize the cell arrest, the K562 cells were incubated for different time periods (0–24 h) with 20 μM of curcumin, allowing a single round of cell division. Then, nuclear alteration or mitotic changes in the treated cells were investigated. To this end, cells were stained with rhodamine-phalloidin and/or DAPI, subsequently they were analyzed by fluorescence microscopy; altered or condensed nuclei were manually counted. Fluorescence microscopy showed that approximately 5% of the cells containing multinucleated chromatin after 24 h of treatment and the number of gigantic cell was not significantly altered (Fig 1B), suggesting that mitosis was arrested in early stages. The results also indicates, that cells containing condensed nuclei increased by up to 10-fold (25% of the cells), after 12 to 24h (Fig 1C), compared with that of the control cells. These results suggested that the arrested cell population comprise cells entering mitosis, rather than G2 phase. In addition, perturbation of the actin cortical rings by curcumin was observed, and this effect was most notable after the 12 h of incubation, when the actin fibers were apparently perturbed and thickened. The actin cytoskeleton network undergoes drastic changes and remodeling during early mitosis; actin network is dismantled and remodeled, resulting in the typical round shape of mitotic cells. Notably, actin fibers were perturbed following 12–18 h of treatment. The actin filaments were shortened and appeared as punctate dots through all cell cytoplasm. During this time, the cells containing condensed nuclei increased also presented tick actin cortical rings (Fig 1C). These results were consistent with the nuclear and cytoplasmic rearrangement occurring in mitosis and the results confirm that mitotic cells were arrested and accumulated following the curcumin treatment.

**Fig 2:**
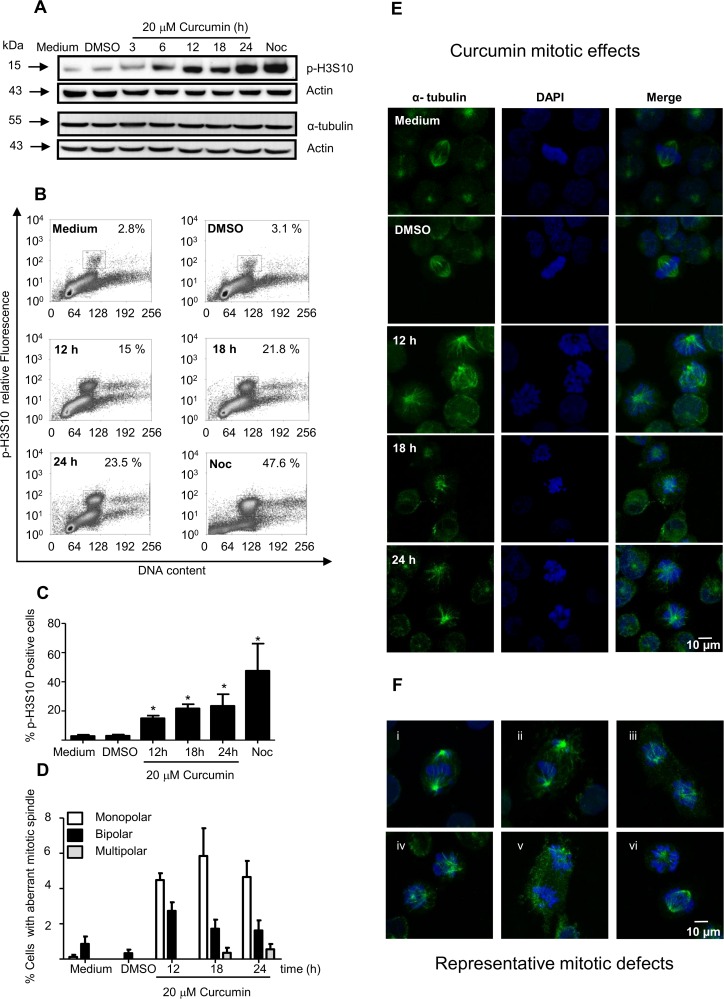
Mitotic spindles in K562 cells were altered by curcumin. **A**) Expression levels of phosphorylsted-histone 3 in serine 10 (p-H3S10) were increased in K562 cells after curcumin treatment, as determined by western blot and **B**) FACS analysis. **C**) The relative numbers of p-H3S10-positive cells are presented as a histogram. K562 cells were stained with DAPI or immunostained using an antibody against α-tubulin (green fluorescence), and the cells with defects in the mitotic spindles were counted. **D**) The percentage of cells with monopolar (white bars), bipolar (black bars) and multipolar (gray bars) nuclei were determined in K562 cells. **E**) Representative fluorescence images of K562 cells bearing mitotic spindle alterations after 12 h, 18 h and 24 h of curcumin treatment are shown. **F**) Images of the most representative mitotic spindle alterations are shown. Actin was used as loading control.

Differential epigenetic markers are contained in the centromeric and pericentromeric chromatin during the chromosome condensation, which occur in the initial phase of mitosis. Phosphorylation of Histone 3 (H3) in serine 10 (p-H3S10) is considered as an early mitosis marker; it is an excellent marker of chromatin condensation and is localized only in pericentromeric heterochromatin. Phosphorylation of p-H3S10 is detected during late G2 phase and remains phosphorylated through the mitosis. Thus, to further analyze this cell arrest; the level of p-H3S10 was measured *in vivo* using FACS analysis or *in vitro* using western blot assays. The results revealed that the p-H3S10 level was increased earlier (after 6 h of treatment), as determined using western blot or FACS analysis, however, not clear change was observed in the level of tubulin by western blot analysis (Fig 2A–2C). These data were consistent with our previous results showed that 35% of K562 cells were arrested in the G2/M, after 6h of the treatment. In addition, to investigate the effect of the curcumin treatment on the tubulin mitotic spindle, treated K562 cells were stained with DAPI and immunostained with anti α-tubulin; the results showed that approximately 8% of the cells present mitotic anomalies, i.e. approximately 25% of the arrested cells. The alterations included monopolar, bipolar and scarcely multipolar spindles (Fig 2D and 2E). Representative mitotic spindle defects are shown (Fig 2F).

**Fig 3:**
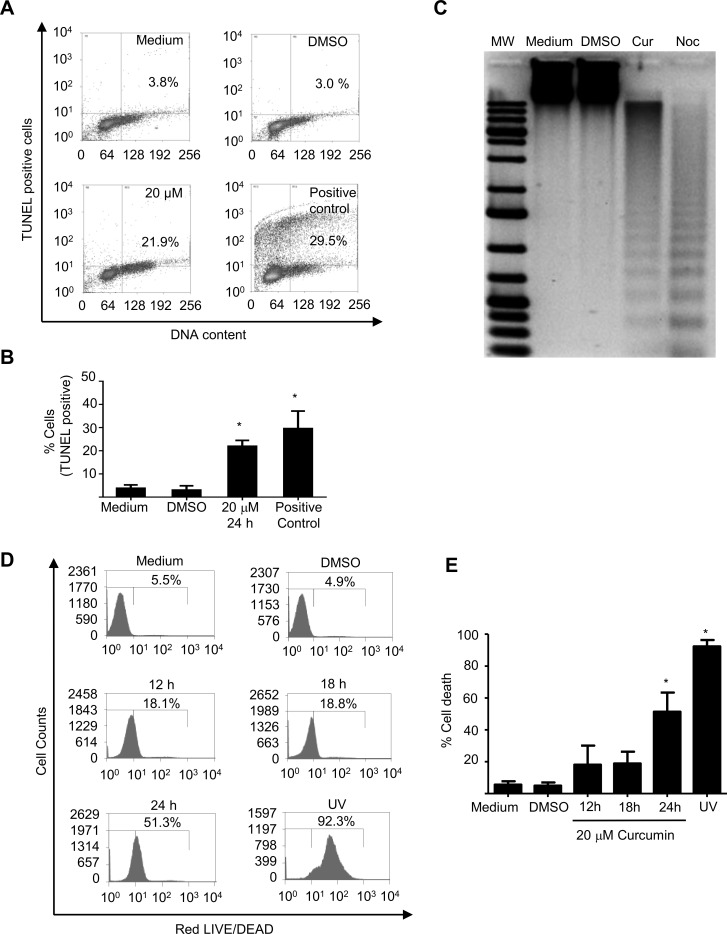
Curcumin induces DNA fragmentation and cell death in K562 cells. **A)** A fraction of the arrested K562 cells treated with 20 μM of curcumin showed DNA damage as indicated by the presence of TUNEL-positive cells and FACS analysis. **B)** The percentage of TUNEL-positive cells is represented by graphic bars, and **C)** production of a typical apoptotic DNA ladder is shown. **D)** Cell death was confirmed by death assays; FACS analysis, and **E)** the results are also shown as graphic bars. As positive controls of DNA fragmentation, we used cells treated with 100 nM nocodazole for 24 h. As a positive control of cell death, K562 cells were exposed to UV (40 mJ/cm^2^) for 2 min in a cross-linker GS Gene linker-UV chamber (Bio-Rad, Hercules CA, USA) and were recovered 24 h post-irradiation.

These results likely meaning that curcumin effect occurs during early event of mitosis, rather than during late mitosis. In contrast, the K562 control cells which were growth with only the DMSO treatment, presented interphasic normal cells and few mitotic cells (1%) with very few metaphasic cells. Notably, the percentage of mitotic cells was lower than expected because up to 50% of these cells were arrested in G2/M after 24h of the treatment (S1 Table). Thus we hypothesize that these cells died during the mitotic arrest; therefore the level of apoptotic cells was further assessed. When the level of apoptosis death was measured by TUNEL analysis; the results revealed that around 25% of these cells (the half of arrested cells) were compromised and presented DNA damage after 24h of the curcumin treatment (Fig 3A and 3B), this observation was supported by the presence of classical apoptotic DNA ladder (Fig 3C). Although, a reduced number of the treated K562 cells died, after 18h of the treatment, cell death increased lately by 3-fold up to 50% at 24h of the treatment, (Fig 3D and 3E), as measured by the life and death assay, indicating that the majority of the arrested cells died after the treatment.

**Fig 4:**
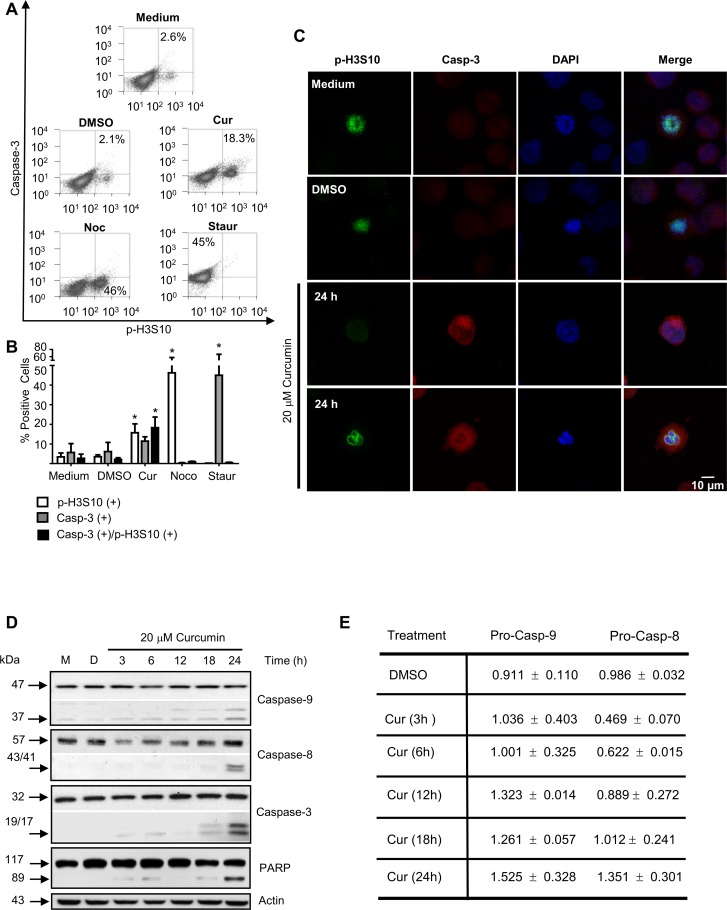
Curcumin induces apoptotic cell death in mitotic K562 cells. **A)** A fraction of the K562 cells arrested with 20 μM of curcumin underwent caspase-dependent apoptosis as revealed by the presence of active caspase-3 in the K562 cells positive for the mitosis p-H3S10 marker and FACS analysis. **B)** The relative numbers of p-H3S10 positive cells with caspase-3 activity are presented as a histogram. **C)** Representative images of the K562 curcumin-treated cells positive for caspase-3 activity using confocal microscopy. **D)** Processed caspases-8, -9 and -3 were detected by western blot analysis and **E)** pro-caspases quantification. Activity was confirmed by cleavage of the caspase-3 substrate PARP, and its protein fragment of 89 kDa was detected. As positive controls for p-H3S10 and caspase activities, K562 cells were exposed for 24h to 100 nM Nocodazole (Noc) or 1 μM Staurosporine (Staur), respectively; M = medium, D = DMSO.

Moreover, cells with condensed chromatin showed active caspase-3 (Fig 4A and 4B); these de data were also confirmed by confocal microscopy (Fig 4C). The processing of caspases -8, -9 and -3 was detected after 18 h of treatment, as shown by western blot analysis. Diminution in the pro-caspase 8 protein level was observed during the first 12 h of the curcumin treatment (Fig 4D), these data were consistent with the cell subpopulation which was killed early by the curcumin treatment (Fig 4E). However, the processing of the caspases in the subpopulation dying during mitotic catastrophe was clearly observed up to 24 h. In addition PARP cleavage confirmed the activation of the caspase pathway (Fig 4D). All these data suggested that apoptosis was triggered by mitotic defects; and apoptosis was likely induced by mitotic catastrophe mechanisms.

**Fig 5:**
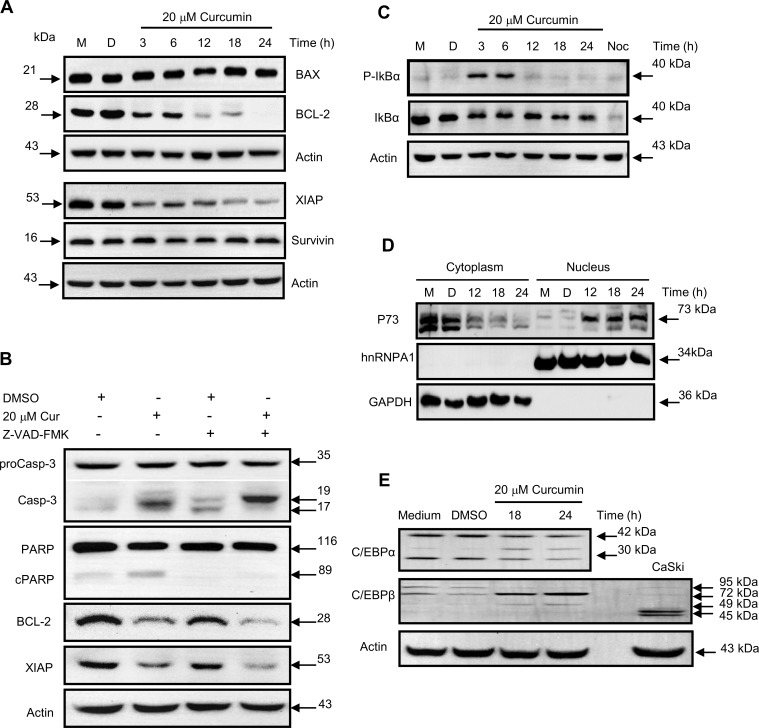
Apoptosis is produced by activation of P73. **A)** Impairment of the expression of the anti-apoptotic proteins BCL-2 and XIAP is shown. **B**) The decreased expression of the anti-apoptotic proteins was caspase independent, as shown by the use of Z-VAD-FMK pancaspase inhibitor. Samples were treated with 2 pulses of 40 μM of pan-caspase inhibitor, Z-VAD-FMK (R&D, Systems), to 12 and 15 h of curcumin treatment. K562 cells were harvested after 18 h, lysed and total protein extracts were obtained for western blot analysis, using specific antibodies against of Caspase-3, PARP, BCL-2 and XIAP. **C**) The diminution of the expression of the antiapoptotic proteins was consistent with decreases in the IκBα and **D**) activation and nuclear translocation of the P73 protein, as consequence of the **E)** diminished expression of the P73 promoter repressor protein C/EBPα; M = medium, D = DMSO.

Finally, to further characterize this apoptosis-like cell death of the K562 cells, which died late after the treatment; the expression of several pro and anti-apoptotic markers was analyzed by western blot analysis. The BCL-2 protein is increased by expression of the BCR-ABL protein hybrid, therefore, BCL-2 was one of the markers selected and examined together with BAX, survivin (BIRC5) and XIAP (BIRC4). The results shown that no expression changes occurs in BAX protein or survivin protein levels; however, the expression of BCL-2 and XIAP proteins was impaired by the curcumin treatment (Fig 5A). Moreover, the addition of the pancaspase inhibitor Z-VAD-FMK, did not affected the decrease in the proteins levels of BCL-2 or XIAP. Conversely, as expected, the 20 kDa protein fragment of the caspase-3 (p20) was accumulated in presence of the pancaspase inhibitor (Fig 5B). Notably, both the BCL-2 and XIAP genes are transcriptionally regulated by NFκB; thus, the activation of the NFκB pathway was also investigated; for this purpose, the level of phosphorylation of IκBα at serines 32 and 36 (S32 and 36) was monitored. S32 and 36 are phosphorylated by IKK promoting IκB degradation, which is necessary for activation of the classical NFκB pathway. The results shown that NFκB was transiently activated early after curcumin treatment, and the phosphorylation level of S32 & 36 of IκBα peaks after 6 h. However, the phosphorylation level of IκBα was very low, following the curcumin treatment for more than 12 h. It was comparable to that presented in the control cells without curcumin stimulus (Fig 5C), this datum was verified by analyzing the nuclear level of Rel A transcription factor (S1 Fig). Moreover, since *P73* gene is responsible for the induction of apoptosis in the absence of *P53* gene expression (K562 cells lack the wild type *P53* gene) the expression of P73 protein was analyzed in nuclear and cytoplasmic protein fractions of the curcumin-treated cells. The results showed that the level of the P73 protein was increased in the nuclear fraction after 12 h of treatment, with a concomitant diminution in the cytoplasmic protein level (Fig 5D). These experiments suggested that the curcumin treatment promotes the nuclear translocation of the P73 protein in K562 cells. P73 protein can trigger apoptosis in cells with mitotic defects. To further check this fact, the level of the active caspase-3 and the fragmentation of its substrate PARP, were analyzed. The results showed a slightly diminution in the level of the active caspase-3 and PARP processing, after treatment with a specific P73 antisense RNA (S2 Fig).

Finally, it has been observed that C/EBPα binds to the P73 promoter and suppresses its transcription; therefore, we hypothesized that the protein levels of C/EBPα would be diminished by the curcumin treatment. The results revealed that expression of C/EBPα was impaired by the treatment. Moreover, the results shown a diminution of two isoforms of the C/EBPα transcription factor, of 30 and 42 kDa; conversely, the expression level of C/EBPβ was increased by the curcumin treatment. Intriguingly, the C/EBPβ protein was not detected as the usual proteins of 45 and 49 kDa (Fig 5E); instead, a protein band with an apparent molecular weight higher than 70 kDa was detected by using the specific commercial antibody. Several reports have shown C/EBPβ sumoylation occurs as a negative regulatory mechanism for cell proliferation and differentiation; the modified C/EBPβ proteins range from the 70–90 kDa [19]. Thus, we hypothesized that this protein might be post-transcriptionally modified in the K562 cells, but this result must be further confirmed. Our data suggested that curcumin treatment produces P73-dependent apoptotic cell death during mitosis and it is triggered by spindle defects and diminution of BCL-2 and XIAP proteins.

## Discussion

Curcumin a biphenolic compound used in Asia as a spice is also a natural chemo-preventive agent for cancer and other diseases [20]. It has been shown to inhibit cell proliferation, cell survival and the production of several cytokines by altering the function of numerous important regulatory pathways, such as NFκB [21] and the transcription factor NRF2^Keap1^ [22]. Moreover, Curcumin targets several different proteins and inhibit the activity of various kinases *in vitro* [23], as well as the activity of histone acetyl transferases [24], therefore, curcumin alters different signaling pathways and also effects epigenetic control of gene expression [25]. Finally, curcumin has been reported to enhance the immune response against cancer cells [26].

Despite extensive studies, the mechanism underlying anti-cancer effects of curcumin are still poorly understood. Several research groups have suggested that curcumin induces cell death in cancer and leukemia chemotherapy-resistant cells; however, the mechanisms by which curcumin induces cell death appear to be different and dose-dependent in various cell types [27]. Curcumin is considered as a potent inducer of apoptosis in cancer cells, mainly by arresting cells in G1 or G2 phase [28–30], but the underlying mechanisms are still unclear. Therefore, a more detailed analysis of the effect of curcumin in cancer and leukemia cells is required for a better understanding of its anti-oncogenic potential.

Curcumin has been reported to disrupt the mitotic spindle structure in different research models such as MCF-7 cells (31) or using mouse 32D progenitor cells transfected with the *BCR-ABL* chimerical cDNA (13). However, in various cell models, curcumin appeared to have different effects on mitosis. In the MCF-7 cells, the treatment with 10 or 20 μM of curcumin causes cell arrest in the G2/M phase; moreover, cells were arrested in the M phase during the first 24–48 h of treatment; the arrested cells had monopolar mitotic spindles which impaired normal chromosome segregation. However, these cells eventually exited the arrest and produce daughter cells with multiple micronuclei, instead of normal daughter cells [31]. Mouse progenitor 32D cells transfected with the *BCR-ABL* chimerical cDNA were used as a chronic myelogenous leukemia progenitor model and were also arrested in the G2/M phase after 18 or 24 h of treatment with curcumin 20μM. The transfected 32D cells were arrested in the M phase and triggering apoptosis.

These cells also had decreased expression of cyclin D2 and cyclin B1 as well as attenuated survivin (BIRC5) expression and mislocalization of Aurora kinase B, the partner of the survivin in the chromosomal passenger complex. These results suggest that apoptosis was triggered by mitotic catastrophe and authors suggest that survivin is a new target of curcumin [13]. However, it is important to note that these two models conserve intact the expression of the P53 protein.

Using a human chronic myelogenous leukemia cell line (K562), we previously observed that after 24 h of treatment with 20 μM of curcumin, one subpopulation of these cells (60%) underwent early apoptosis, but other subpopulation was arrested and accumulated in the G2/M phase of the cell cycle; suggesting that K562 cells contain different cell populations, which differentially trigger programmed cell death. In the surviving cells (40%), approximately half were arrested in G2/M phase. The arrested cells underwent apoptosis until 18 h of the treatment, and cells death peaked after 24 h. These data was confirmed because caspases-3 and -8 were detected in its active form, after 18 h of the treatment. Notably, caspase-9 was activated earlier, suggesting that apoptosis was triggered by the intrinsic mechanism. The arrested cells showed alterations in the mitotic apparatus, and mitotic spindles collapse in a few cells, but monopolar mitotic cells and micronuclei were also detected, suggesting that spindle collapse occurred in prometaphase. It is possible that a few surviving cells accumulates chromosome alterations, however experiments did not progress past 24 h, to ensure that there was only one cell division, and therefore, no accumulation of chromosome segregation anomalies were observed. Thus, this point should be addressed in the future, because only 50% of the treated cells were dead at 24h.

Our research model differs from the mouse progenitor model using 32D cells transfected with BCR-ABL, because no impairment of cyclin B1 in the curcumin-treated K562 cells was observed. In addition, no changes in survivin protein levels were observed. Notably, survivin is phosphorylated early in promethapase by the mitotic kinase CDK1, and is stabilized in the Chromosomal Passenger Complex; this protects survivin from proteasomal degradation until metaphase [32] and likely preserves its apoptotic role during early mitosis failure. Our data showed mitotic alterations similar to those of the MCF7 cells treated with 20 μM curcumin [31], but not those of the mouse 32D cells, although, MCF7 lacks of the expression of the *BCR-ABL* fused gene. All those results suggested that the curcumin effects are independent of the expression of the BCR-ABL hybrid protein, as was previously found in the cells 32D model. Interestingly, the K562 cells have a disrupted expression of the *P53* gene [33], whereas MCF7 cells and progenitor mouse cells 32D cells have a wild type P53. Thus, some of the differences in the response might be due to lack of wild type p53 protein expression.

**Fig 6:**
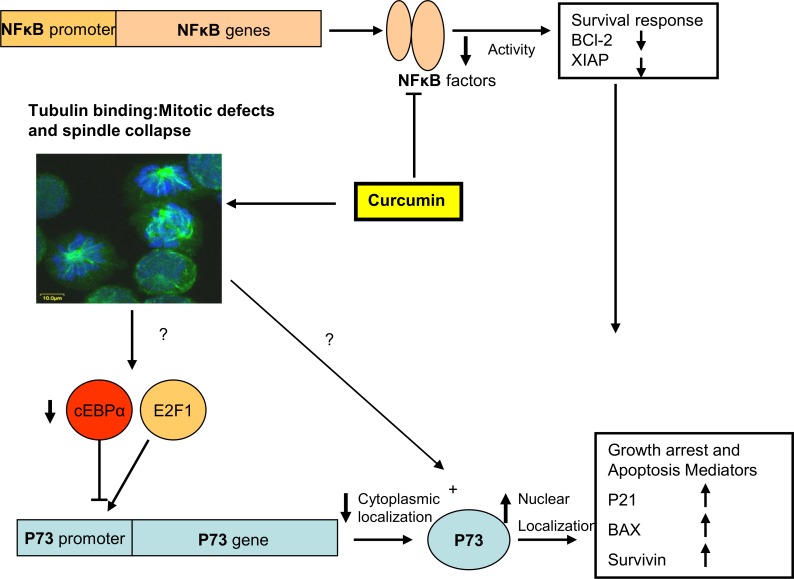
Schematic representation of the mitotic catastrophe and P73-dependent apoptosis mechanisms triggered by curcumin treatment in the K562 cells.

Because some K562 cells died after long exposure to curcumin, we characterized this subpopulation by using molecular and cellular tools. Under this conditions, up to 50% of the surviving cells were arrested in the G2/M phase. The analysis of the curcumin-arrested cells by using rodhamine-phalloidine and confocal microscopy showed mitotic cells with thickening of the actin cortical ring; this remodeling of actomyosin fibers is typically observed during normal mitosis [34]. In addition, using specific antibodies against tubulin, we observed that approximately 8% of the arrested cells showed altered chromatid separation and different mitotic defects. After, 18 h of treatment caspases activities were detected by using fluorescent substrates; and caspase-3 activity was confirmed by PARP cleavage and apoptosis was confirmed by DNA fragmentation or formation of DNA ladder. Additionally, active caspase-3 was observed in mitotic cells, as shown by co-localization of the active caspase-3 and a mitotic marker (p-H3S10). Apoptosis was produced by increased expression of nuclear P73 and a transitory activation of the NFκB activity that was incapable to sustain the expression level of the anti-apoptotic proteins, BCL-2 [35] and XIAP [36]. In addition, it was previously reported that a novel amonafide analogue (an isoquinoline derivative) activates the E2F1/P73 pathway in K562 cells, which were arrested in G2/M and underwent apoptosis [37]. The increased expression of P73 was produced by impairment of the C/EBPα expression, which is consistent with previous reports, where it was observed that C/EBPα bound to promoter of P73 and suppressed its transcription [38]. Therefore, the protein level of C/EBPα is diminished in conditions when P73 is expressed (see schematic representation in Fig 6). Notably, that was previously reported that shikonine a natural naphtoquinone produces an increase in P73 protein level concomitant with the diminution of BCL-2 protein level, in MCF7 and HeLa cells [39]. Finally, the nuclear expression of the P73 in K562 cells is important because this gene is responsible for the induction of apoptosis in the absence of P53 [40]; K562 cells lacks the wild type *P53* gene; thus, the expression and activation of the P73 protein acts as regulator of apoptosis, similar to P53.

In summary, curcumin treatment might produce a P73-dependent apoptotic cell death in chronic myelogenous leukemia cells (K562), which was triggered by mitotic catastrophe, due to sustained BAX and survivin expression and impairment of the anti-apoptotic proteins BCL-2 and XIAP.

## Supporting Information

S1 Fig Active Rel A (NFkB p65), is diminished in nuclear compartment after 12 h of the curcumin treatment. The level of NFκB p65/Rel A was investigated in cytoplasm and nuclear of the K652 cells treated with 20 μM curcumin during 6 or 12 h, using western blot analysis. The level of hnRNPA1 and GAPDH proteins was analyzed as control for the purity of the nuclear and cytoplasmic fractions.
(TIF)

S2 Fig Apoptosis cell death induced by P73 during mitotic catastrophe. **A)** diminution of BIM mRNA level in K562 after curcumin treatment as analyzed by RT-PCR. Cells were incubated with 20μM curcumin for 12, 18 or 24 h; then harvested and total RNA was isolated and use for first strand cDNA synthesis. **B)** Level of P73 protein of K562 cells transfected with 50 nM of Ctrl siRNA-A (sc-37007) or 50 nM P73 siRNA (sc-36167); cells were harvested after 24 h post-transfection, lysed and analyzed by western blot by using specific P73 antibody or. **C)** or specific antibodies against active caspases-9 and -3 or PARP, the 89 kDa cleaved fragment of PARP (Asp 214) is also shown. Actin was used as loading control.
(TIF)

S1 Table Comparation of the percentage of K562 cells arrested in G2/M phase of the cell cycle or killed after treatment with 20 μM or 30 μM curcumin. (TIF)
