# Supplementary figures and images for: A Subpopulation of the K562 Cells Are Killed by Curcumin Treatment after G2/M Arrest and Mitotic Catastrophe
Source: PLoS One. 2016 Nov 10;11(11):e0165971. doi: 10.1371/journal.pone.0165971 (PMC5104431; doi:10.1371/journal.pone.0165971)

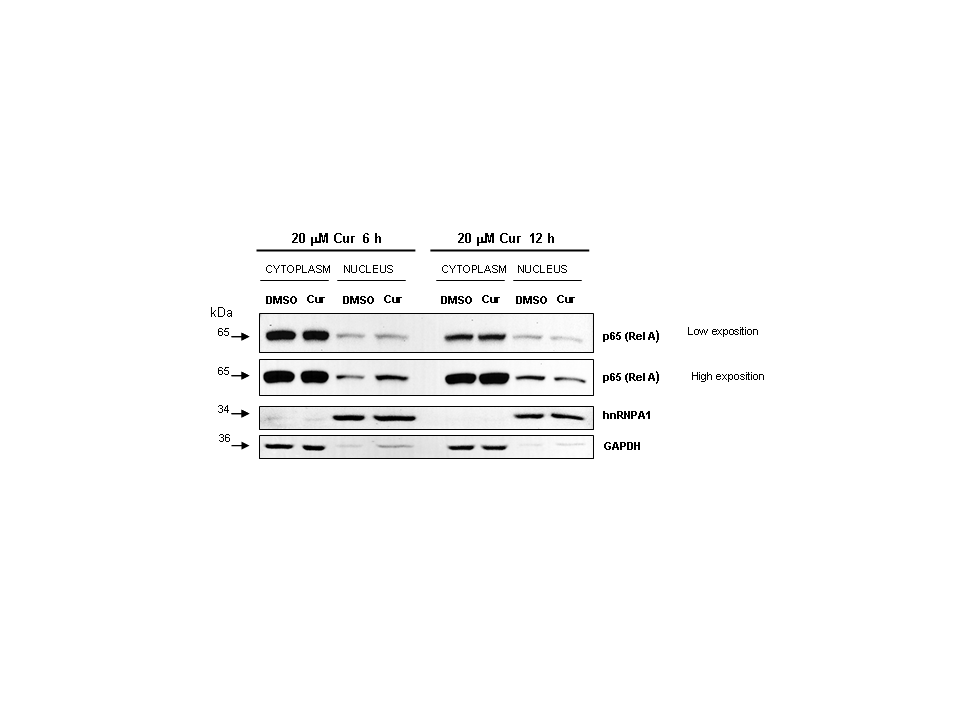

Supplement: S1 Fig — The level of NFκB p65/Rel A was investigated in cytoplasm and nuclear of the K652 cells treated with 20 μM curcumin during 6 or 12 h, using western blot analysis. The level of hnRNPA1 and GAPDH proteins was analyzed as control for the purity of the nuclear and cytoplasmic fractions. (TIF) [file pone.0165971.s001.tif]

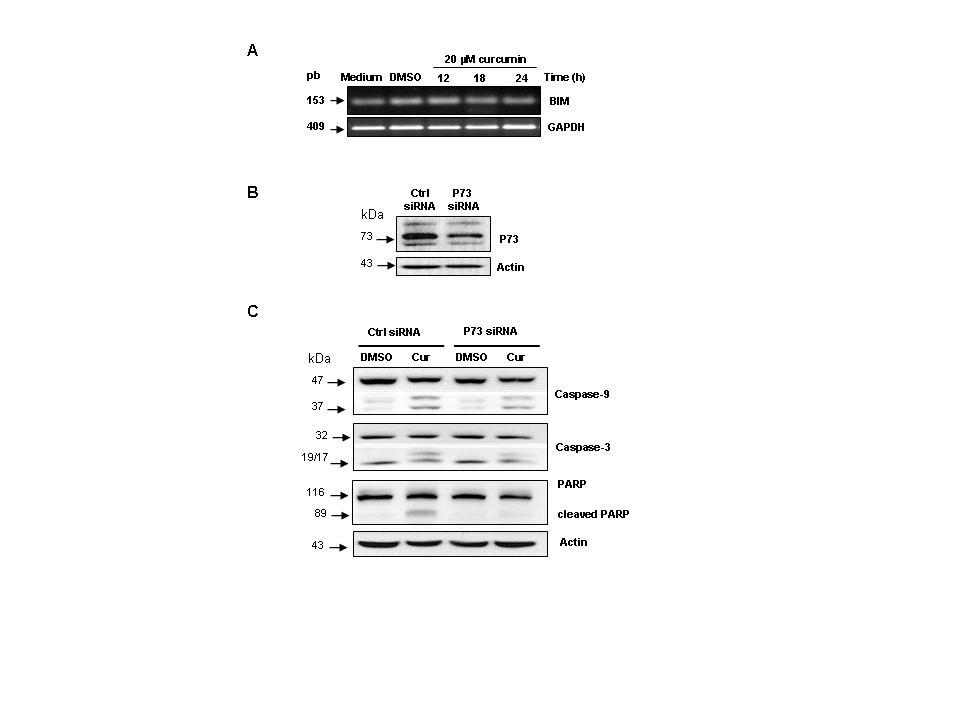

Supplement: S2 Fig — A) diminution of BIM mRNA level in K562 after curcumin treatment as analyzed by RT-PCR. Cells were incubated with 20μM curcumin for 12, 18 or 24 h; then harvested and total RNA was isolated and use for first strand cDNA synthesis. B) Level of P73 protein of K562 cells transfected with 50 nM of Ctrl siRNA-A (sc-37007) or 50 nM P73 siRNA (sc-36167); cells were harvested after 24 h post-transfection, lysed and analyzed by western blot by using specific P73 antibody or. C) or specific antibodies against active caspases-9 and -3 or PARP, the 89 kDa cleaved fragment of PARP (Asp 214) is also shown. Actin was used as loading control. (TIF) [file pone.0165971.s002.tif]

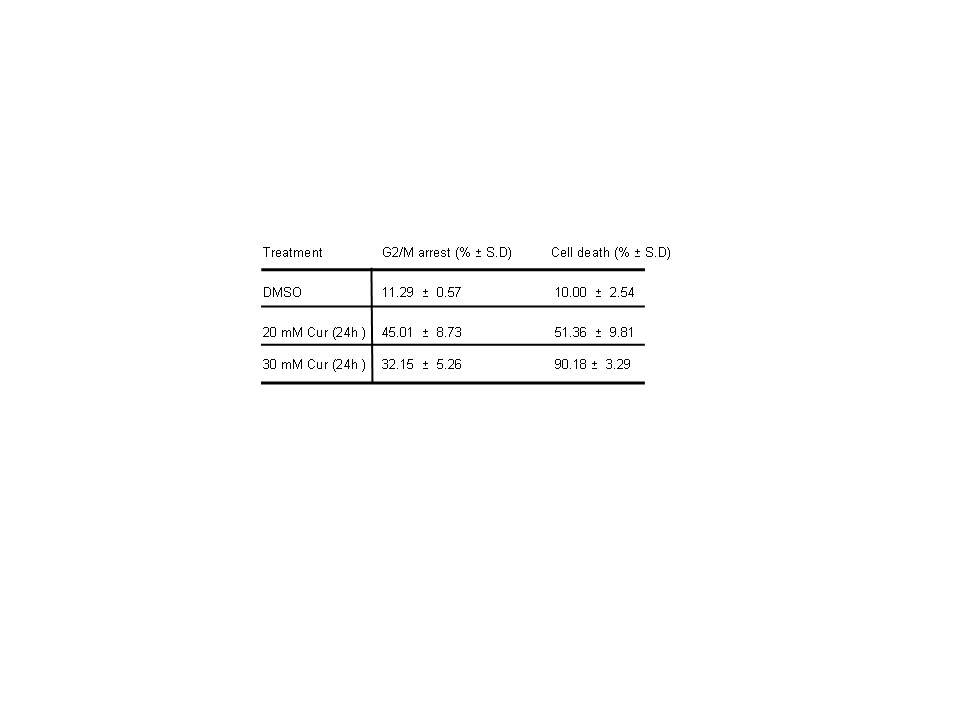

Supplement: S1 Table — (TIF) [file pone.0165971.s003.tif]
